# IL-6 deficiency attenuates p53 protein accumulation in aged male mouse hippocampus

**DOI:** 10.1007/s10522-019-09841-2

**Published:** 2019-10-09

**Authors:** Izabela Bialuk, Magdalena Cieślińska, Oksana Kowalczuk, Tomasz A. Bonda, Jacek Nikliński, Maria M. Winnicka

**Affiliations:** 1 Department of General and Experimental Pathology, Medical University of Białystok, Mickiewicza 2c, 15-222 Białystok, Poland; 2 Department of Clinical Molecular Biology, Medical University of Białystok, Waszyngtona 13, 15-269 Białystok, Poland

**Keywords:** IL-6 deficiency, p53, Hippocampus, Apoptosis, Autophagy

## Abstract

Our earlier studies demonstrated slower age-related memory decline in IL-6-deficient than in control mice. Therefore, in the present study we evaluated the effect of IL-6 deficiency and aging on expression of p53, connected with accumulation of age-related cellular damages, in hippocampus of 4- and 24-month-old IL-6-deficient C57BL/6J (IL-6KO) and wild type control (WT) mice. The accumulation of p53 protein in hippocampus of aged IL-6KO mice was significantly lower than in aged WT ones, while *p53* mRNA level was significantly higher in IL-6-deficient mice, what indicates that the effect was independent on *p53* transcription. Presence of few apoptotic cells in hippocampal dentate gyrus and lack of changes in levels of pro-apoptotic Bax, antiapoptotic Bcl-2, as well as in p21 protein in aged animals of both genotypes, points to low transcriptional activity of p53, especially in aged WT mice. Because the amount of p53 protein did not correlate with the level of Mdm2 protein, its main negative regulator, other than Mdm2-dependent mechanism was involved in p53 build-up. Significantly higher mRNA levels of autophagy-associated genes: *Pten, Tsc2,* and *Dram1* in IL-6KO mice, in conjunction with significantly lower amount of Bcl-2 protein in 4-month-old IL-6KO mice, suggests that lack of IL-6/STAT3/Bcl-2 signaling could account for better autophagy performance in these mice, preventing excessive accumulation of proteins. Taken together, attenuated p53 protein build-up, absence of enhanced apoptosis, and transcriptional up-regulation of autophagy-associated genes imply that IL-6 deficiency may protect hippocampus from age-related accumulation of cellular damages.

**Electronic supplementary material:**

The online version of this article (10.1007/s10522-019-09841-2) contains supplementary material, which is available to authorized users.

## Introduction

Interleukin 6 (IL-6) is a small signaling glycoprotein with a diverse set of actions that depend on the target cell type. It has been shown that, in the central nervous system (CNS) it regulates neuronal and synaptic functions, as well as behavior (Erta et al. 2012; Gruol 2015). Within the CNS IL-6 is mainly synthesized by astrocytes, and to a lesser extent by microglia and neurons (Gruol 2015). In basal conditions IL-6 mRNA and protein are expressed in limited amounts in several brain regions (Aniszewska et al. 2015; Gadient and Otten 1994; Schobitz et al. 1993). IL-6 exerts biological effects either via the membrane bound IL-6 receptor (classical signaling) or via the soluble form of IL-6 receptor (trans-signaling), and the latter one has been suggested to be of particular importance in the CNS (Erta et al. 2012; Heinrich et al. 2003; Spooren et al. 2011). Both signaling pathways depend on the membrane bound gp130 subunits. Formation of the IL-6/IL-6R/gp130 complex phosphorylates tyrosine kinases of the Janus kinase (JAK) family and triggers a sequence of events resulting in gp130 phosphorylation, followed by activation of the signal transducer and activator of transcription-3 (STAT3). In addition to activation of the STAT3 pathway, the RAS/mitogen-activated protein kinase (MAPK), phosphatidylinositol-3 kinase (PI3 K) and insulin receptor substrate (IRS) pathways can be activated by the IL-6/IL-6R/gp130 complex (Boulanger et al. 2003; Erta et al. 2012; Heinrich et al. 2003).

Aging is a natural process with gradual decline of many normal biological functions of cells such as: DNA repair, regulation of cell proliferation, and immune response (Feng et al. 2007). Long lived neuronal cells are likely to accumulate mutations in the DNA leading to impaired cellular functions. Moreover, neurons are characterised by high metabolic activity and high consumption of oxygen. Therefore, these cells are exposed to higher levels of oxidative stress in comparison with other cell types (Best 2009). DNA damage that exceeds a threshold is associated with apoptosis or senescence (Best 2009; Brady and Attardi 2010). Importantly, increasing with age and in neurodegenerative diseases IL-6 expression was recognized as an accelerator of senescence (Erschler 1993; Godbout and Johnson 2004; Tha et al. 2000). Under stressful conditions such as inflammation, brain injury and certain CNS diseases IL-6 level significantly raises, both on the periphery and in the CNS (Erta et al. 2012; Gruol 2015). Increased expression of IL-6 in normal aging (Godbout and Johnson 2004; Marsland et al. 2006; Weaver et al. 2002), augmented in certain neurodegenerative diseases, has been shown to interfere with cognitive functions (Bermejo et al. 2008; Cacquevel et al. 2004; Maggio et al. 2006; McAfoose and Baune 2009; Müller et al. 1998; Luterman et al. 2000; Trapero and Cauli 2014). Importantly, GFAP-IL-6 transgenic mice, in which elevated levels of IL-6 in the CNS are produced by astrocytes, exhibited progressive behavioural, physiological, as well as anatomical abnormalities, particularly in hippocampus and cerebellum, developing earlier in homozygote than in heterozygote mice (Campbell et al. 1998; Heyser et al. 1997; Gruol 2015).

A tumor suppressor, p53 protein, is long-recognized to suppress cancer through the induction of cell-cycle-arrest or apoptosis in response to different cellular stress signals (Brady and Attardi 2010). However, studies have demonstrated that function of p53 extends beyond the capacity to trigger cell-cycle arrest and programmed-cell death, and novel activities, such as the regulation of metabolism, autophagy and the oxidative status of the cell, are emerging (Brady and Attardi 2010; Chumakov 2007; Rufini et al. 2013).

The present study expanded our earlier experiments, performed on 4- and 24-month-old IL-6-deficient mice, assessing the influence of IL-6 deficiency on cognitive processes. In the previous study we demonstrated the attenuation of learning ability in Morris water maze (Bialuk et al. 2018), as well as the attenuation of recognition memory in IL-6KO young adult mice (Bialuk et al. 2019) in comparison to IL-6 producing mice. However, age-related progression of these alterations was slower in IL-6KO group than in controls. Moreover, IL-6-deficient mice demonstrated better retrieval of acquired information, more pronounced when delay between learning completion and testing was longer (Bialuk et al. 2018; Bialuk and Winnicka 2018). Because age-related accumulation of cellular damages resulting in increased p53 expression (Brady and Attardi 2010) may be involved in age-related memory decline we investigated the effect of IL-6 deficiency and aging on p53 protein abundance in hippocampus, a key structure for learning and memory processes.

## Materials and methods

All procedures were approved by the Local Animal Ethics Committee in Białystok, Poland and were performed in compliance with the European Communities Council Directive 2010/63/EU. Naïve, male 4-month-old (young adult) and 24-month-old (aged) IL-6-deficient mice C57BL/6J^IL-6−/−TMKopf^ (IL-6KO) and reference wild type (WT) animals (C57BL/6J), originally purchased from the Jackson Laboratory (USA), were obtained from the Centre for Experimental Medicine of the Medical University of Białystok. The mice were maintained in a temperature-controlled environment (22 ± 1 °C), humidity (45–55%), with a 12 h light–dark cycles beginning at 7 a.m. and were housed in polycarbonate cages, five animals per cage, with water and commercial food (Labofeed H Standard, Morawski, Poland) available ad libitum. Mice were sacrificed by cervical dislocation. No sedation was used. Brains were immediately excised manually and transferred into the 10% phosphate-buffered formalin. Subsequently, brains were processed into the paraffin blocks collected for histological and immunohistological examination. For molecular biology analyses hippocampi taken from mice after excision, under the × 3-magnifying glass, were immediately placed in sterile Eppendorf tube, subsequently frozen in liquid nitrogen directly and stored in − 80 °C until further procedure. One, randomly taken left or right hippocampus was used for Western blot, while the other for quantitative Real-Time PCR. Genotype of mice was confirmed by polymerase chain reaction as described previously (Bonda et al. 2013).

### Western blot

Hippocampus was homogenized in an ice-cold RIPA buffer (Sigma) containing protease and phosphatase inhibitors (Sigma), using a hand homogenizer. The homogenates were centrifuged at 4 °C for 10 min at 8000 rpm. The protein content in the supernatant was measured using Bradford method (Bio-Rad). Samples were frozen at − 80 °C until further analysis. Protein homogenates were subjected to SDS-PAGE according to the method of Laemmli in 15% polyacrylamide gel and blotted onto nitrocellulose membranes 0.2 mm (BioRad). Membranes were blocked with 5% BSA (Sigma) or 5% not fat dry milk for 1 h at room temperature (RT). Primary antibodies recognizing mouse’s Bax (Cell Signaling, 2772S, 1:500), Bcl-2 (Cell Signaling, 2870S, 1:1000), p21 (ThermoFisher, PA1-30399, 1:1000), p53 (BD Pharmingen, 554166, 1:1000), Mdm2 (Thermo Scientific, PA5–27209, 1:2000), GFAP (Abcam, ab7260, 1:10000), and α–Tubulin (Santa Cruz Biotechnology, sc-5286, 1:8000), were used. Secondary antibodies were conjugated with horseradish peroxidase (anti Rabbit IgG-HRP, AbD Serotec—STAR54; anti Mouse IgG-HRP, Sigma–A9303). Blots were visualized using enhanced chemiluminescence reaction (Thermo Scientific) and exposed on the x-ray film (X-Omat Blue, Carestream). Scanned films were quantified using the Image J software (National Institutes of Health, USA). The results of particular experiments were related to the expression of proteins in the control group, which was set as 1.

### Immunofluorescence

The sections after deparaffinization and rehydration, were pre-treated with proteinase K solution (1:800) and blocked with 10% donkey serum in phosphate buffered saline (PBS), pH 7.4, for 1 h at RT. The primary antibody against GFAP (ab7260, Abcam) was applied at 1:500 dilution in PBS for 90 min at RT. Next, the sections were washed and incubated with secondary antibody conjugated with biotin (Donkey Anti-Rabbit IgG, 711-065-152, Jackson Immuno Research Laboratories) at a 1:200 in PBS dilution for 1 h at RT, followed by washing with PBS containing Tween 20. Subsequently, the sections were incubated with streptavidin-Alexa Fluor® 488 (S32354, Life Technologies) at a dilution of 1:1000 in PBS for 40 min at RT in the dark, washed and counterstained with HOECHST 33258 (Sigma-Aldrich**)** in PBS for 2 min. at RT in the dark. Slides were cover slipped in Dako Mounting Medium and evaluated using fluorescence microscope (Olympus BX 41), using Olympus UPlanFLN 40 ×/0.75 and Olympus PlanCN 20 ×/0.40 objective.

### TUNEL method

After deparaffinization the sections were subjected to the fluorescein Terminal deoxynucleotidyl transferase mediated dUTP-marker Nick-End Labelling (TUNEL) using ApopTag® Fluorescein In Situ Apoptosis Detection Kit (Millipore, S7110) according to the manufacturer procedure. Counterstaining of cell nuclei was performed using HOECHST 33258 (Sigma-Aldrich**)**. All slides were analysed and photographed using fluorescence microscope (Olympus BX 41).

### RNA isolation and quantitative real-time polymerase chain reaction (qRT-PCR)

Homogenization of hippocampus was performed using TissueLyser (Qiagen) for 2 min. at 30 Hz with one Stainless Steel Bead, 5 mm (Qiagen, Cat No. 69989). Total RNA was isolated using Rneasy Lipid Tissue Mini Kit (Qiagen, Cat. No. 74804) and QIAcube apparatus (Qiagen) according to the manufacturer’s protocols. RNA was dissolved in 40 μl of RNase-free water. Its quantity was evaluated using NanoDrop 2000c Spectrophotometer (Thermo Fisher Scientific, Inc., Wilmington, DE, USA) immediately after isolation. RNA quality, including 28S/18S ratio and RNA integrity number (RIN) was assessed with 2100 Bioanalyzer System (Serial. No. DE72905449) and an RNA 6000 Nano Kit (Agilent Technologies Inc., Santa Clara, CA, USA, cat. no 5067-1511) according to the manufacturer’s protocol. 500 ng of total RNA was transcribed into cDNA using RT2 First Strand Kit (Qiagen, Cat. No. 330404) in Labcycler (Model No. 1120240193; SensQuest GmbH, Göttingen, Germany) according to the manufacturer’s protocol. cDNA was stored in − 80 °C until qRT-PCR using RT2 SYBR Green qPCR Mastermix (Qiagen, Cat. No. 330502) and Custom RT2 PCR Array plates (Qiagen, Cat. No. 330171). Information regarding primers used in the assay is presented in Table 1. The amplification reaction was performed in 25-μl reaction mixture. Each sample was run in duplicates. The qRT-PCR cycling conditions were as follows: first cycle—95 °C for 10 min, followed by 45 cycles: 95 °C for 15 s and 60 °C for 1 min. RT-qPCR was performed in Roche LightCycler 480 apparatus with software for evaluation of baseline and cycle threshold (Ct). The presence of a single peak at the melting temperature for each gene was confirmed by melting curves inspection. Expression level was quantified as Ct values normalized for the mean of the two reference control genes transferrin receptor (*Tfrc*) and phosphoglycerate kinase 1 (*Pgk1*) (Boda et al. 2009) using equation: ΔCt = Ct_group_ − Ct_ref_. Fold-change (FC) in the mRNA level was calculated as FC = 2^−ΔΔCt^, where ΔΔCt equals the difference between the normalized expression of the gene in the IL-6KO mice (Ct_IL-6KO_) and its normalized expression in the corresponding age-matched WT animal (Ct_WT_) (IL-6 vs. WT ones) (Schmittgen and Livak 2008). When the difference in expression within genotype was calculated young adult group of appropriate genotypes was taken as the control (24- vs. 4-month-old). For the statistical analyses, logarithmically transformed FC values were used (log2(FC)).

**Table 1:** List of primers used in qRT-PCR

Gene symbol	Refseq #	Official full name	Qiagen catalog number
*Il6*	NM_031168	Interleukin 6	PPM03015A
*Trp53*	NM_001127233	Transformation related protein 53	PPM02931C
*Mdm2*	NM_010786	Transformed mouse 3T3 cell double minute 2	PPM02929C
*Tsc2*	NM_001039363	Tuberous sclerosis 2	PPM27785A
*Sesn1*	NM_001013370	Sestrin 1	PPM04988B
*Pten*	NM_008960	Phosphatase and tensin homolog	PPM03379A
*Dram1*	NM_027878	DNA-damage regulated autophagy modulator 1	PPM28326A
*Tfrc*	NM_011638	Transferrin receptor	PPM03499C
*B2m*	NM_009735	Beta-2 microglobulin	PPM03562A
*Pgk1*	NM_008828	Phosphoglycerate kinase 1	PPM03700A
GDC		Mouse genomic DNA contamination control	PPM65836A
PPC		Positive PCR control	PPX63339
RTC		Reverse transcription control	PPX63340

### Statistics

Statistical analyses were performed using Statistica 13.0 and GraphPad Prism 5. All data were first assessed for normality. Due to some asymmetric distribution of data, indicated by Shapiro–Wilk test, results from Western blot were analysed by analysis of variance (ANOVA) with Bonferroni post hoc test or by Kruskal–Wallis with Dunn’s multiple comparison post hoc test, when appropriate. The difference in the mRNA expression levels were analysed with paired Wilcoxon signed rank test. Western blot protein abundance and ΔCT values from qRT-PCR were subjected to General Linear Model (GLM) to assess the effect of genotype, age and their interaction. In all instances *p* < 0.05 was considered a statistically significant difference.

## Results

### p53 and Mdm2 expression

**Fig. 1:**
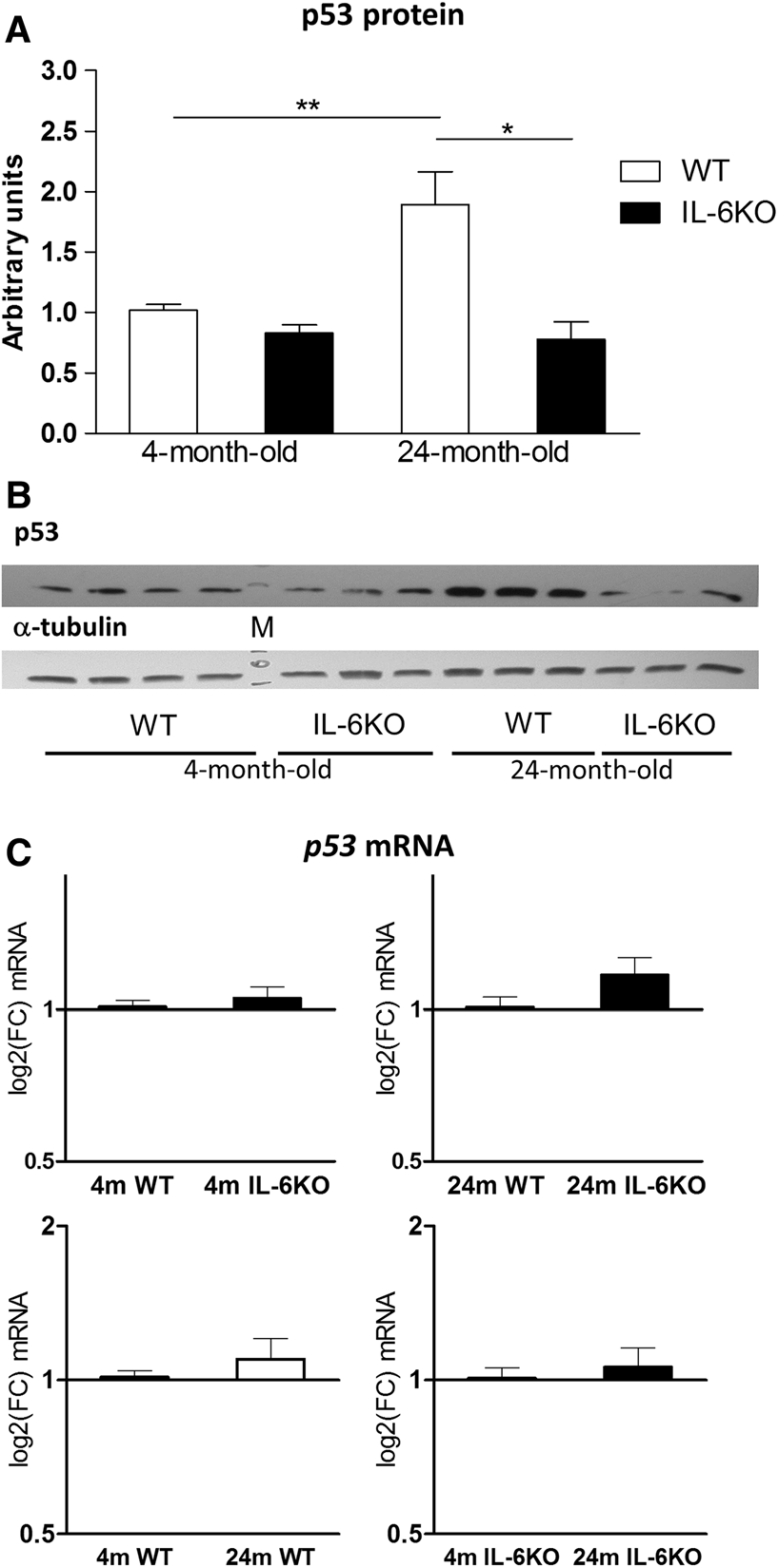
Amount of p53 protein (**a**) and its mRNA transcripts (**c**) in hippocampus of 4- and 24-month-old IL-6-deficient (IL-6KO) and wild type control (WT) mice. Level of mRNA expression was defined as log2(FC), where FC stands for fold-change difference in mRNA level between indicated groups. Bars represent mean ± SEM obtained from six animals in each group. Expression of p53 protein was low and comparable in 4-month-old groups of both genotypes. In 24-month-old WT mice the amount of p53 protein increased significantly in comparison with 4-month-old WT (**p < 0.01) and 24-month-old IL-6KO (*p < 0.05) animals (Kruskal–Wallis with Dunn’s post hoc test). According to GLM the accumulation of p53 protein was both genotype- and age-dependent (*p* < 0.005 and *p* < 0.05, respectively). **c** There were no significant differences in the *p53* mRNA levels between four groups of mice, however, according to GLM analysis the *p53* mRNA level turned out to be influenced by genotype (*p* < 0.05). **b** Representative immunoblot for p53 protein is shown together with α-tubulin as a loading control. *M* molecular weight marker

The amount of p53 protein in hippocampus of young adult groups was low and comparable, while in aged animals it increased only in WT ones (Fig. 1a). Evaluation with Kruskal–Wallis test yielded H(4,24) = 13.78, *p* < 0.05 and Dunn’s post hoc test revealed significant increase of p53 protein amount in aged WT mice in comparison with 4-month-old WT and 24-month-old IL-6KO mice (*p* < 0.01 and *p* < 0.05, respectively). In aged IL-6KO mice the level of p53 protein was similar to IL-6KO young adult ones. Analysis of *p53* mRNA expression in hippocampal cells revealed higher level of its transcript in 24-month-old IL-6KO mice than in age-matched WT animals, but the difference was insignificant. In 4-month-old IL-6KO mice the level of *p53* mRNA was only slightly higher than in age-matched WT ones, as well as in both aged groups in comparison with the respective young adult group (Fig. 1c). GLM analysis revealed significant influences of genotype and age on parameters assessed in Western blot and in qRT-PCR. Abundance of p53 protein was both genotype- and age-dependent (Table 2, *p* = 0.0011 for genotype, *p* = 0.0283 for age, and *p* = 0.0099 for genotype*age interaction), while the amount of *p53* mRNA transcript turned out to be only genotype-dependent (Table 3, *p* = 0.0359).

**Table 2:** Effects of genotype and age on hippocampal protein abundance evaluated by Western blot in 4- and 24-month-old IL-6-deficient (IL-6KO) and wild type control (WT) mice

Parameter	Groups (mean ± SEM), n = 12				*p* values		
Protein abundance	WT	IL-6KO	Young adult (4 m)	Aged (24 m)	Genotype	Age	Genotype*age interaction
p53	1.4553 ± 0.1866	0.8130 ± 0.0832	0.9447 ± 0.0473	1.3346 ± 0.2237	**0.0011**	**0.0283**	**0.0099**
Mdm2	1.0357 ± 0.0542	0.9402 ± 0.0653	1.0963 ± 0.0485	0.8791 ± 0.0558	> 0.05	**0.0063**	> 0.05
p21	0.8995 ± 0.0817	0.8848 ± 0.0774	0.9322 ± 0.0704	0.8521 ±0.0862	> 0.05	> 0.05	> 0.05
Bax	1.0303 ± 0.0834	0.8740 ± 0.0798	0.9582 ± 0.0636	0.9461 ± 0.1019	> 0.05	> 0.05	> 0.05
Bcl-2	0.9230 ± 0.0433	0.7593 ± 0.0270	0.8737 ± 0.0362	0.8086 ± 0.0490	> 0.05	> 0.05	> 0.05
GFAP	2.0029 ± 0.3326	2.3315 ± 0.4991	1.6729 ± 0.1920	2.6067 ± 0.5042	> 0.05	> 0.05	> 0.05

Bold *p* values indicate significant influence of a given factor according to General Linear Model (GLM)

**Table 3:** Effects of genotype and age on hippocampal mRNA transcript levels measured by qRT-PCR in 4- and 24-month-old IL-6-deficient (IL-6KO) and wild type control (WT) mice

Parameter	Groups (mean ΔCT ± SEM), n = 12				*p* values		
ΔCT for gene	WT	IL-6KO	Young adult (4 m)	Aged (24 m)	Genotype	Age	Genotype*age interaction
*p53*	4.7332 ± 0.0582	4.4588 ± 0.0602	4.6615 ± 0.0642	4.6254 ± 0.0605	**0.0359**	> 0.05	> 0.05
*Mdm2*	1.5632 ± 0.0519	1.3695 ± 0.0408	1.3547 ± 0.0392	1.5773 ± 0.0505	**0.0026**	**0.0006**	> 0.05
*Pten*	− 0.4502 ± 0.0837	− 0.7827 ± 0.0473	− 0.7470 ± 0.0706	− 0.4843 ± 0.0736	**0.0008**	**0.0076**	> 0.05
*Tsc2*	1.7806 ± 0.1240	1.2768 ± 0.0586	1.4002 ± 0.1118	1.6626 ± 0.0753	**0.0007**	> 0.05	> 0.05
*Sesn1*	4.0593 ± 0.1033	3.8600 ± 0.0759	3.7620 ± 0.0921	4.1530 ± 0.1058	> 0.05	**0.0020**	> 0.05
*Dram1*	6.9689 ± 0.1003	6.6295 ± 0.0717	6.7184 ± 0.0948	6.9795 ± 00793	**0.0385**	> 0.05	> 0.05

Bold *p* values indicate significant influence of a given factor according to General Linear Model (GLM)

**Fig. 2:**
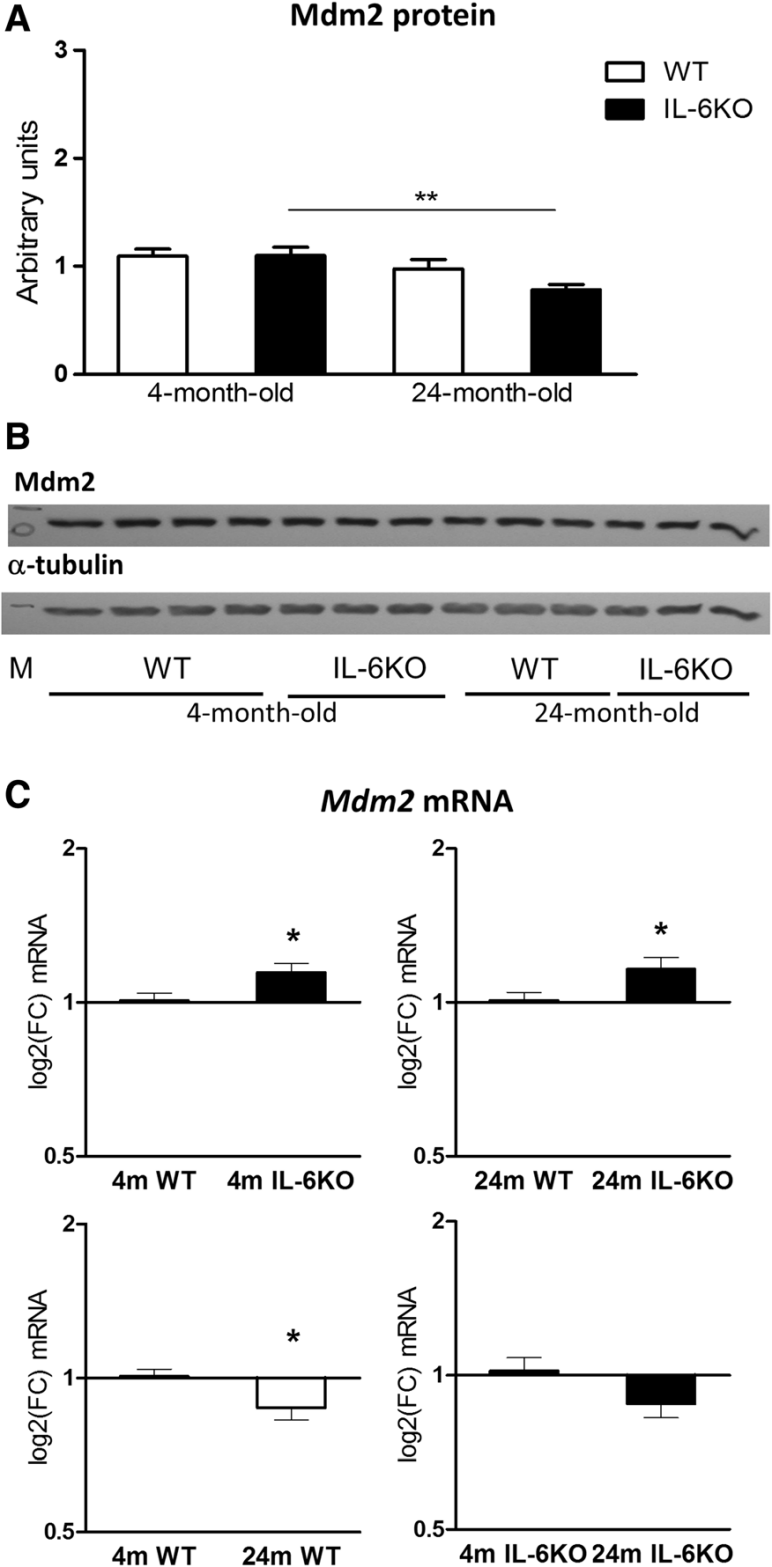
Amount of Mdm2 protein (**a**) and its mRNA transcripts (**c**) in hippocampus of 4- and 24-month-old IL-6-deficient (IL-6KO) and wild type control (WT) mice. Level of mRNA expression was defined as log2(FC), where FC stands for fold-change difference in mRNA level between indicated groups. Bars represent mean ± SEM obtained from six animals in each group. There were no significant differences in the Mdm2 protein level in young adult mice, and aging was associated with decrease in its amount, which was statistically significant in IL-6-deficient mice in comparison with age-matched WT controls (**p < 0.01, ANOVA with Bonferroni post hoc). GLM analysis revealed significant influence of age on the Mdm2 protein abundance (*p* < 0.001). (**c**) In both 4- and 24-month-old IL-6-deficient mice *Mdm2* mRNA level was significantly higher in comparison with respective control WT animals (*p < 0.05), and aging was associated with significant decrease in *Mdm2* mRNA in WT controls (*p < 0.05, Wilcoxon signed rank test). According to GLM analysis the expression of *Mdm2* mRNA was both genotype- and age-dependent (*p* < 0.005 and *p* < 0.001, respectively). **b** Representative immunoblot for Mdm2 protein is shown together with α-tubulin as a loading control. *M* molecular weight marker

To determine whether increase in p53 protein level, resulted from diminished action of its main negative regulator, the Mdm2 protein was examined. Analysis of Mdm2 Western blot quantitation revealed no differences in its amount between young adult groups (Fig. 2a). In aged mice the level of Mdm2 was moderately decreased in IL-6KO mice and slightly decreased in WT animals (Fig. 2a). ANOVA of Mdm2 protein amount yielded F(3,20) = 4.336, *p* = 0.0165 and Bonferroni post hoc test revealed significantly decreased Mdm2 protein level in 24-month-old IL-6KO mice in comparison with 4-month-old IL-6-deficient ones (*p* < 0.05). Comparison of four groups revealed that *Mdm2* mRNA expression was higher in both IL-6KO than in respective WT groups, and lower in both aged groups in comparison with genotype-matched young adult groups (Fig. 2c). Wilcoxon signed rank test revealed significantly higher level of *Mdm2* transcripts in 4- and 24-month-old IL-6KO mice than in age-matched WT animals (*p* < 0.05) and significantly lower level of *Mdm2* transcripts in 24-month-old than in 4-month-old WT animals (*p* < 0.05). When genotype and age effect on Mdm2 abundance was assessed by GLM, significant differences were found for age factor regarding Mdm2 protein levels (Table 2, *p* = 0.0063) and for genotype and age regarding *Mdm2* mRNA transcript amounts (Table 3, *p* = 0.0026 and *p* = 0.0006, respectively).

### p21 protein

Expression of p21 protein, a mediator of p53-dependent cell-cycle arrest, was examined to determine the potential consequences of age-associated increase in p53 protein level. The amount of p21 protein was comparable in all tested groups, indicating that neither IL-6 deficiency, nor aging affected its expression in hippocampal cells (Supplementary material Fig. S1A). GLM analysis showed lack of significant effects of either genotype, age or their interaction on p21 protein level (Table 2).

### Apoptosis and its markers

Because increased level of p53 protein may suggest enhanced apoptosis, the abundance of its markers: Bax and Bcl-2 proteins was evaluated. The amount of both proteins was lower in 4- and 24-month-old IL-6KO mice in comparison with respective WT groups (Supplementary material Fig. S2). ANOVA of Bcl-2 protein amount yielded F(3,20) = 0.0218, *p* = 4.016, and Bonferroni post hoc test revealed significant difference (*p* < 0.05) only between 4-month-old IL-6KO and WT animals (Supplementary material Fig. S2B). Moreover, in both genotypes aging had no effect on Bax and Bcl-2 protein level. Similarly, as for p21, GLM analysis showed lack of significant effects of either genotype, age or their interaction on Bax and Bcl-2 protein levels (Table 2).

**Fig. 3:**
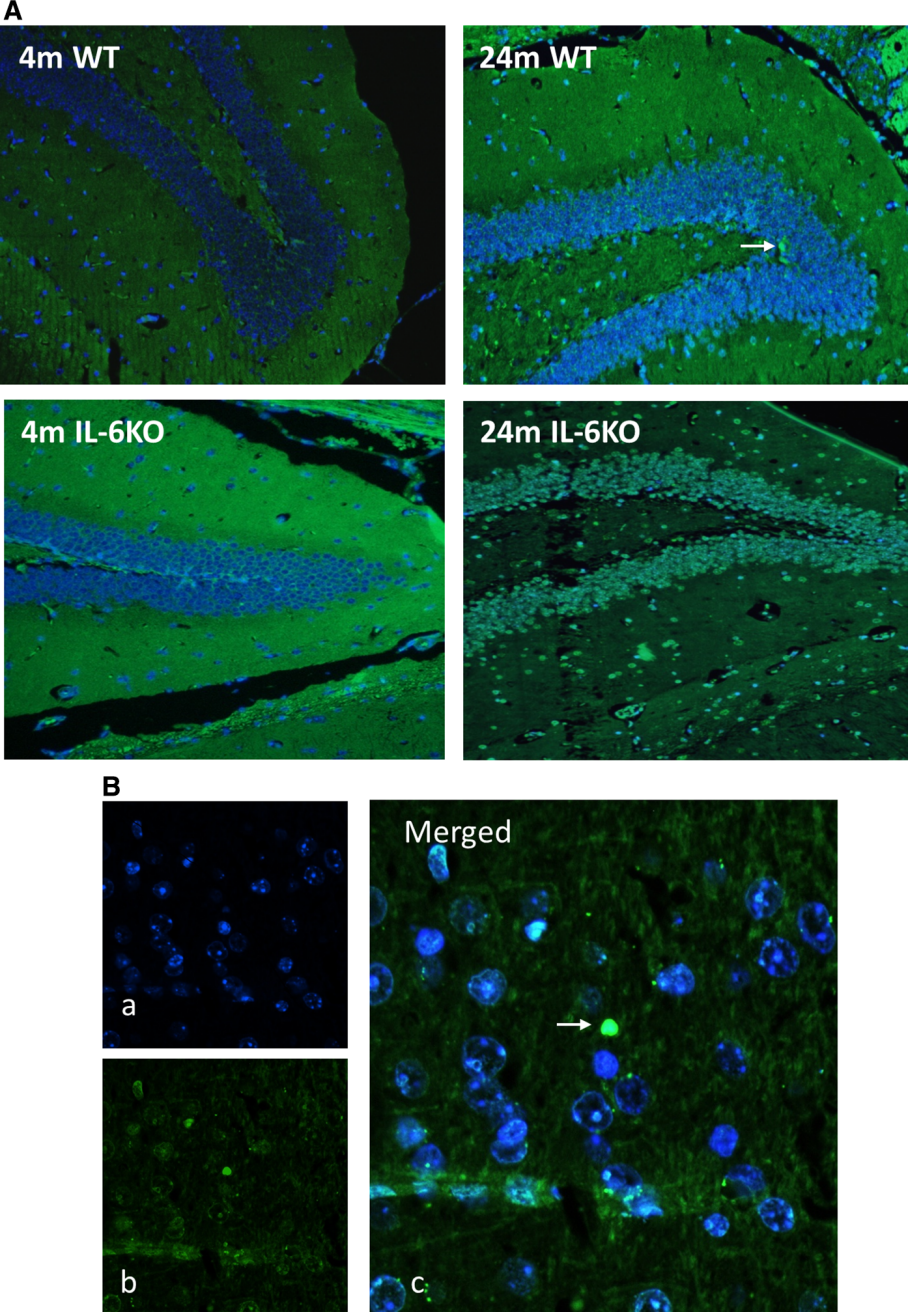
**a** Representative microphotographs of Hoechst 33258 and TUNEL staining of hippocampal dentate gyrus form 4- and 24-month-old IL-6-deficient (IL-6KO) and wild type control (WT) mice revealed single apoptotic cells in hippocampus of aged mice of both genotypes, (magnification × 200). **b** (a) Fluorescent Hoechst 33258 (blue) nuclear staining, (b) TUNEL staining (green) indicating apoptotic nucleus, (c) Merged images of Hoechst 33258 (blue) and TUNEL (green) staining, (magnification × 400). White arrow indicates apoptotic cell

To confirm results obtained from molecular studies visualisation of apoptotic cells using TUNEL method was performed on hippocampal slices. Microscopic examination of dentate gyrus in 12 sections (2 from 6 animals in each group) showed presence of only single apoptotic cells in hippocampal dentate gyrus of both aged groups indicating that programmed cell-death was not enhanced (Fig. 3a).

### GFAP and IL-6 mRNA

**Fig. 4:**
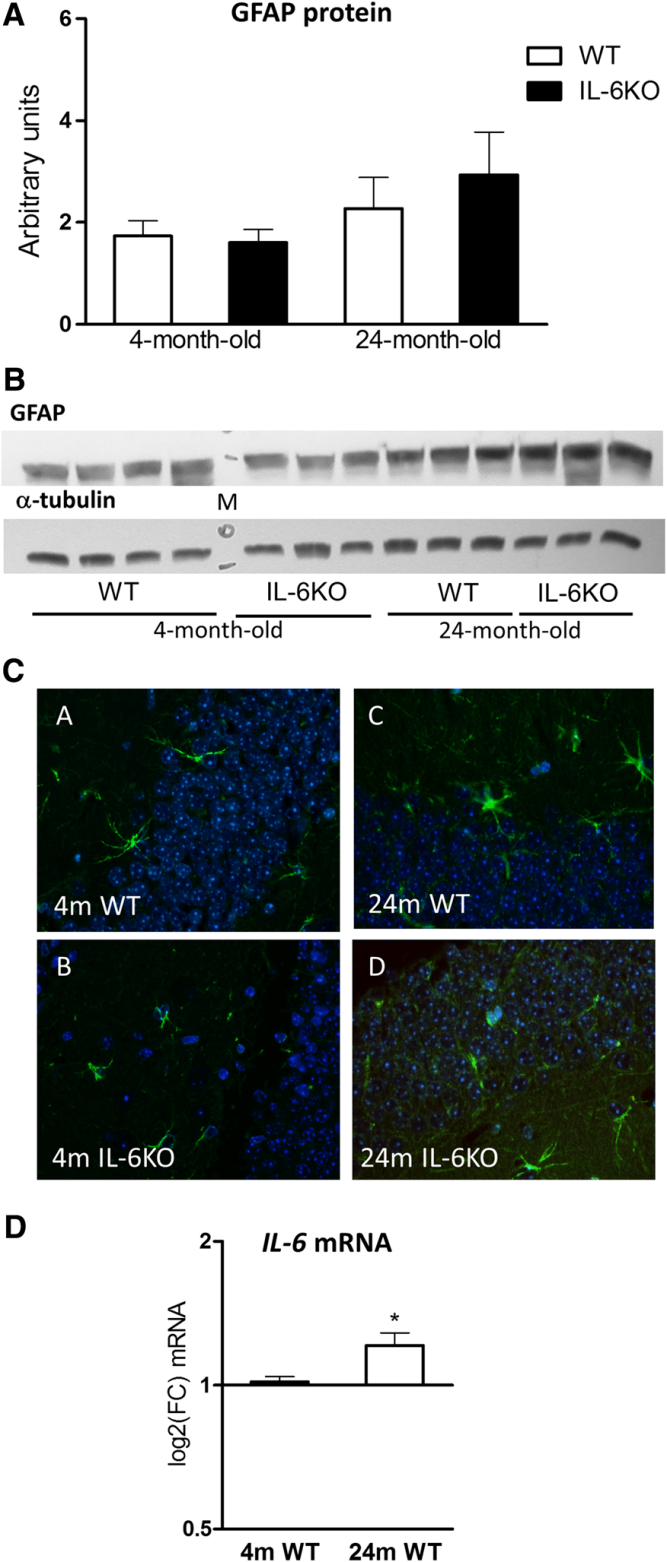
**a** Amount of glial fibrillary acidic protein (GFAP) in hippocampus of 4- and 24-month-old IL-6-deficient (IL-6KO) and wild type control (WT) mice was comparable in both young adult groups and insignificantly higher in both aged groups. Bars represent mean ± SEM obtained from six animals in each group. **b** Representative immunoblot for GFAP protein is shown together with α-tubulin as a loading control. *M* molecular weight marker. **c** Tissue staining of GFAP, an astrocytic marker (green), showed similar intensity in both 4-month-old groups and its moderate increase in both 24-month-old groups (magnification, × 20), **d***IL*-*6* mRNA in hippocampus was significantly higher in 24-month-old WT mice in comparison with 4-month-old WT ones (*p < 0.05, Wilcoxon signed rank test). Level of mRNA expression was defined as log2(FC), where FC stands for fold-change difference in mRNA level

Because aging is associated with hyperplasia and hypertrophy of glial cells, the major source of IL-6 in CNS, evaluation of glial cell abundance was performed by Western blot protein quantification and by tissue staining with antibody directed against present in astrocytes glial fibrillary acidic protein (GFAP). Although the GFAP protein level was higher in both aged than in respective young adult groups the differences were insignificant (Fig. 4a). Tissue staining of GFAP showed its comparable intensity in hippocampal dentate gyrus of both young adult groups and its mild increase in aged IL-6KO and WT animals (Fig. 4c). GLM evaluation, however, revealed lack of significant influence of age, genotype or their interaction regarding the abundance of GFAP in this brain structure (Table 2).

Analysis of *IL*-*6* mRNA quantitation revealed its significant up-regulation in 24-month-old WT mice in comparison with 4-month-old WT animals (*p* = 0.0455, Wilcoxon signed rank test). Although, the difference in the mRNA for IL-6 was statistically significant, the amount of this cytokine in aged WT mice increased by about 35% in comparison with younger animals (Fig. 4d).

### mRNA quantitation of selected p53-dependent genes

**Fig. 5:**
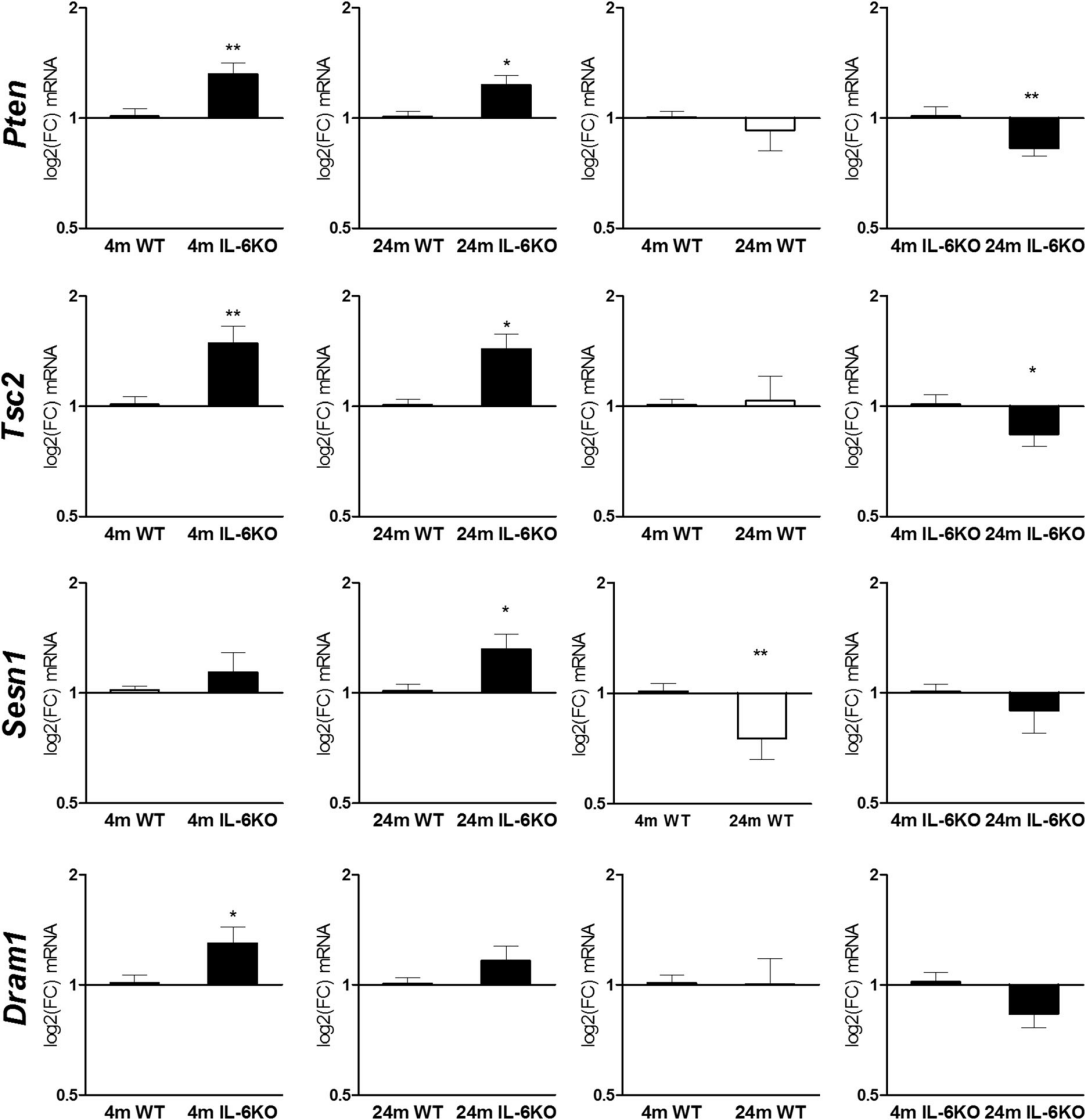
mRNA transcript levels of *Pten, Tsc2*, *Sesn1* and *Dram1* genes in hippocampus of 4- and 24-month-old IL-6-deficient (IL-6KO) and wild type control (WT) mice. Level of mRNA expression was defined as log2(FC), where FC stands for fold-change difference in mRNA level between indicated groups. Bars represent mean ± SEM obtained from six animals in each group. Levels of *Pten* mRNA and *Tsc2* mRNA were significantly higher in both 4- and 24-month-old IL-6KO mice in comparison with age-matched WT controls (**p < 0.01, and *p < 0.05, respectively). Aging was associated with significant decrease in *Pten* mRNA and *Tsc2* mRNA levels (**p < 0.01, and *p < 0.05, respectively) in IL-6KO animals (Wilcoxon signed rank test). IL-6 deficiency was associated with statistically significant increase in *Sesn1* mRNA in aged animals in comparison with respective WT group (*p < 0.05), while aging was associated with significantly decreased its mRNA transcript in WT control mice (**p < 0.01, Wilcoxon signed rank test). Deficiency of IL-6 resulted in significantly higher expression of *Dram1* mRNA in 4-month-old mice (*p < 0.05), while aging diminished *Dram1* mRNA level, but the effect was insignificant (Wilcoxon signed rank test). GLM analysis revealed influence of genotype on the transcription of *Pten*, *Tsc2,* and *Dram1* (*p *< 0.0005*, p *<0.0005 and *p *<0.05, respectively), and influence of age on the transcription of *Pten* and *Dram1 (p *<0.01 and *p *<0.005, respectively)

Results of qRT-PCR are presented on Fig. 5. Statistical evaluation of Phosphatase and tensin homologue (*Pten*) mRNA level by Wilcoxon signed rank test revealed significantly higher expression of *Pten* in both 4- and 24-month-old IL-6-deficient mice in comparison with age-matched control WT mice (*p* = 0.0020 and *p* = 0.0161, respectively). Aging was associated with down-regulation of *Pten* mRNA expression, which was statistically significant in 24-month-old IL-6KO mice in comparison with 4-month-old IL-6KO ones (*p* = 0.00149, Wilcoxon signed rank test).

Analysis of Tuberos sclerosis 2 (*Tsc2*) mRNA showed significantly higher expression of its transcript in both 4- and 24-month-old IL-6KO mice in comparison with age-matched WT controls (*p* = 0.0022 and *p* = 0.0269, respectively, Wilcoxon signed rank test). Evaluation of *Tsc2* mRNA expression within genotypes showed significant decrease in *Tsc2* transcript in 24-month-old IL-6KO mice in comparison with younger ones (*p* = 0.0137, Wilcoxon signed rank test).

Also, Sestrin 1 (*Sesn1*) mRNA expression was higher in IL-6KO mice than in respective WT groups. Statistical analysis with Wilcoxon signed rank test revealed that significant difference was only between 24-month-old IL-6KO and WT group (*p* = 0.049). Moreover, aging was associated with decrease in *Sesn1* mRNA expression, which was insignificant in IL-6KO and significant in WT mice (*p* = 0.0098) in comparison with appropriate genotype-matched younger group.

Analysis of Damage-regulated autophagy modulator 1 (*Dram1*) mRNA quantification revealed that in both young adult and aged IL-6KO mice the level of *Dram1* transcript was higher in comparison with age-matched WT animals, however the difference was significant only between young adult groups of mice (*p* = 0.0322, Wilcoxon signed rank test). Aging was associated with insignificant decrease in *Dram1* mRNA expression in IL-6KO mice, but not in WT ones, in which it remained on similar to 4-month-old WT mice level.

GLM analysis revealed significant influence of genotype and/or age on the amount of mRNA transcripts for selected genes (Table 3). Transcription of *Pten* (*p* = 0.0008), *Tsc2* (*p* = 0.007), and *Dram1* (*p* = 0.0385) was influenced by genotype. Moreover, transcription of *Pten* (*p* = 0.0076) and *Sesn1* (*p* = 0.0020) was influenced by age factor.

## Discussion

Our study revealed significantly attenuated accumulation of p53 protein in hippocampus of 24-month-old mice with IL-6 deficiency. Because in 4-month-old IL-6KO and age-matched WT mice the p53 protein levels were low and comparable, it may indicate that significant increase in p53 protein abundance only in WT mice was associated with higher level of IL-6 in senescent animals. Accumulation of p53 protein in WT animals was significantly higher while in IL-6KO mice it did not change overtime. Since in IL-6KO mice significantly higher expression of *p53* mRNA was not accompanied by an increase in the amount of p53 protein in these animals it indicates that attenuation of p53 protein accumulation in aged IL-6KO mice was independent on *p53* gene transcription. Similar lack of substantial increase in *p53* mRNA levels in aged mice was also described by others. Edwards et al. (2007) evaluated *p53* mRNA levels in 5- and 25-month-old C57BL/6J mice in whole brain homogenates and reported lack of significant difference in *p53* gene expression between young and old brains.

The *p53* gene becomes activated in response to myriad cellular stress signals. In cells under potent stress p53 triggers irreversible programs of apoptosis or senescence, while under conditions of mild stress, the same protein elicits protective, pro-survival action to maintain genome integrity and viability in cells with reparable damages. The exact cell fate is dependent on the cell type, environmental milieu and the nature of stress (Brady and Attardi 2010). High level of p53 protein suggests potent stress. Upon DNA damage or other stressors activation of p53 leads to a transient expression the cyclin-dependent kinase inhibitor (CKI) p21. Subsequently, this either triggers G1 cell cycle arrest or leads to a chronic state of senescence or to apoptosis (Georgakilas et al. 2017). Our data demonstrated lack of differences in the amount of p21 between IL-6-deficient and IL-6-producing mice, indicating that increased p53 protein amount in hippocampus of aged WT mice did not affect p21 protein expression. Further evaluation of programmed cell-death markers: pro-apoptotic Bax and anti-apoptotic Bcl-2 revealed that neither Bax, nor Bcl-2 did not show significant differences in the amount of protein when compared between genotypes of aged animals. Observed lack of changes in the apoptotic markers in senescent animals was in accordance with presence of single apoptotic cells detected in hippocampus of aging mice of both genotypes, confirming that increased level of p53 protein was not associated with enhanced apoptosis. Despite higher p53 accumulation in hippocampus of aged WT animals lack of changes in the expression of its down-stream protein targets may suggest the attenuation of its transcriptional activity. Posttranslational modifications such as phosphorylation and acetylation have been associated with increased, while methylation with decreased, p53 protein transcriptional activity (Ivanov et al. 2007). Importantly, in our study increased level of p53 protein observed in aged control WT mice did not affect the expression of p21, Bax, and Bcl-2 proteins. Therefore, lack of significant effects of high p53 protein level on its cellular targets in aged WT mice may indicate that methylated p53 form constituted its majority. Moreover, our previous study evaluating significance of IL-6 deficiency in the mouse myocardium showed suppression of p53 protein accumulation in aged IL-6KO mice in comparison with WT ones, and that increased amount of p53 protein in the cardiomyocytes of aged mice expressing endogenous IL-6 constituted a cytoplasmic pool (Bonda et al. 2019). This may explain why increased accumulation of p53 protein in aged animals was not accompanied by concurrent increase of its transcriptional activity.

Significantly lower level of p53 protein in aged IL-6KO than in WT mice, may point to the involvement of IL-6 in mechanisms regulating the amount of p53 protein in hippocampal cells. A major mechanism regulating p53 protein levels involves a reciprocal relationship with murine double minute 2 (Mdm2) protein. Under normal conditions Mdm2 binds with p53 protein blocking its activity and promoting transport for ubiquitin–proteasome degradation. Variety of cellular stressors have been shown to stabilize p53 protein via Mdm2 degradation and transcription of p53 target genes (Engel et al. 2007). In the current study aged IL-6KO mice demonstrated significantly decreased Mdm2 level in comparison with young adult ones, while in aged WT mice the Mdm2 was on the same level when compared with younger controls. Similarly, in our previous study performed on the mouse myocardium the expression of Mdm2 protein was lower in aged IL-6KO in comparison with young adult mice, whereas in WT control animals an age-related decline in Mdm2 was less pronounced and did not reach the statistical threshold (Bonda et al. 2019). In both studies the abundance of Mdm2 was at the same level in young adult groups. Regarding *Mdm2* mRNA, its level was significantly higher in IL-6KO mice than in WT mice, in both age groups but it was not followed by a higher amount of protein product, while aging was associated with decreased *Mdm2* mRNA expression in both genotypes, followed by only slight in WT, and significant diminution of its protein amount in IL-6KO animals. Because the absence of endogenous IL-6 was accompanied by lower amount of p53 protein in hippocampus, not dependent on its ubiquitin–proteasome degradation, it may suggest that IL-6 is involved in other mechanisms responsible for p53 accumulation. Therefore, we evaluated the expression of selected genes involved in autophagy, especially that multiple p53 target genes have been shown to influence this process. On one hand p53-mediated transcriptional up-regulation of AMPK, Pten, and sestrins has been demonstrated to activate autophagy. On the other hand, cytoplasmic p53 has been shown to suppress autophagic flux through an unknown mechanism (Rufini et al. 2013). Moreover, it has been also shown that IL-6 influences autophagy through both inhibitory and stimulatory action (Qin et al. 2015). IL-6/STAT3 signaling pathway was demonstrated to inhibit autophagy in U937 cells, while it activated this process in pancreatic cancer cells (Kang et al. 2012). In our setting IL-6 deficiency was associated with up-regulation of autophagy-related genes: *Pten, Tsc2, Sesn1,* and *Dram1*, which was more pronounced in young adult mice. Moreover, in 4-month-old IL-6-deficient mice the amount of Bcl-2 protein, which is suggested to take part in IL-6-dependent autophagy regulation, was significantly decreased in comparison with age-matched WT controls. Taking into consideration that in young adult IL-6KO mice p53 protein level was low and expression of *Pten, Tsc2, Sesn1,* as well as *Dram1* was upregulated it may suggest that lack of IL-6/STAT3/Bcl-2 signaling could account for better autophagy performance. In aged animals of both genotypes the expression levels of assessed autophagy-related genes were lower in comparison with respective young adult groups what may lead to an increased accumulation of altered protein forms.

In the CNS IL-6 is mainly synthesized by astrocytes (Erta et al. 2012; Gruol 2015) and under stressful conditions these cells may become a significant source of reactive oxygen species (ROS) affecting function of neurons. Senescence and aging are associated with an increase in the level of oxidative-damaged proteins, lipids and DNA (Rufini et al. 2013), and aging astrocytes have been shown to present an increased mitochondrial oxidative metabolism leading to an age-dependent increase in hydrogen peroxide generation and NFκB signalling in the cytosol, as well as to its translocation to the nucleus (Jiang and Cadenas 2014). Because GFAP protein is a reliable astrocytic marker we compared its amount in young adult and aged animals of both genotypes. The moderate increase in the intensity of GFAP staining in both aged groups, was in accordance with 35% increase of *IL*-*6* mRNA amount in hippocampus of aged WT animals. Therefore, increased under normal condition synthesis of IL-6 could account for rather low-level of age-related oxidative stress and this weak genotoxic stress was insufficient to activate apoptosis. However, diminished level of p53 protein in hippocampus of aged mice not producing endogenous IL-6 might be associated with slower progression of age-related changes. Moreover, higher expression of genes associated with autophagy in IL-6KO mice points to the involvement of IL-6 in age-related accumulation of cellular damages in the hippocampus.

## Electronic supplementary material

Below is the link to the electronic supplementary material.

Supplementary material 1 (PDF 153 kb)
