# Supplementary material for: IL-6 deficiency attenuates p53 protein accumulation in aged male mouse hippocampus
Source: Biogerontology. 2019 Oct 9;21(1):29–43. doi: 10.1007/s10522-019-09841-2 (PMC6942598; doi:10.1007/s10522-019-09841-2)
Supplement: Supplementary file 1 — Supplementary material 1 (PDF 153 kb) [file 10522_2019_9841_MOESM1_ESM.pdf]

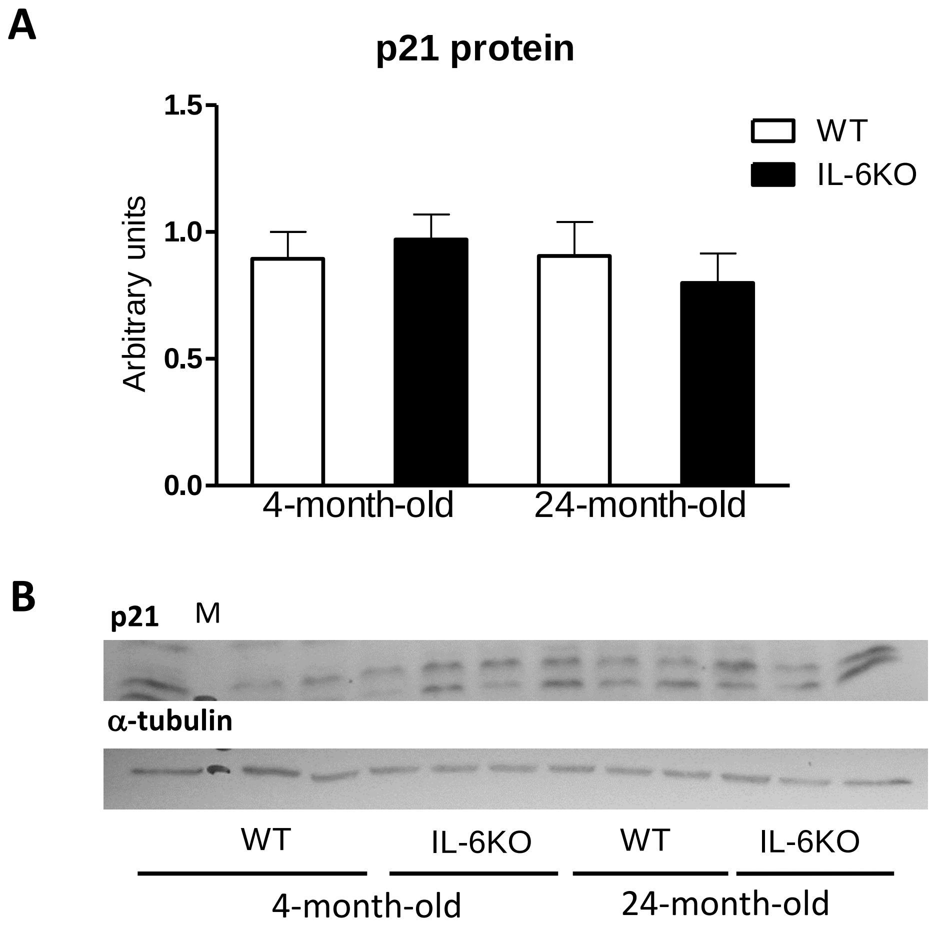

Fig. S1 p21 protein level (**A**) in hippocampus of 4- and 24-month-old IL-6-deficient (IL-6KO) and wild type control (WT) mice was comparable in all tested groups. Bars represent mean ± SEM obtained from 6 animals in each group. (**B**) Representative immunoblot for p21 protein is shown together with α-tubulin as a loading control. M - molecular weight marker

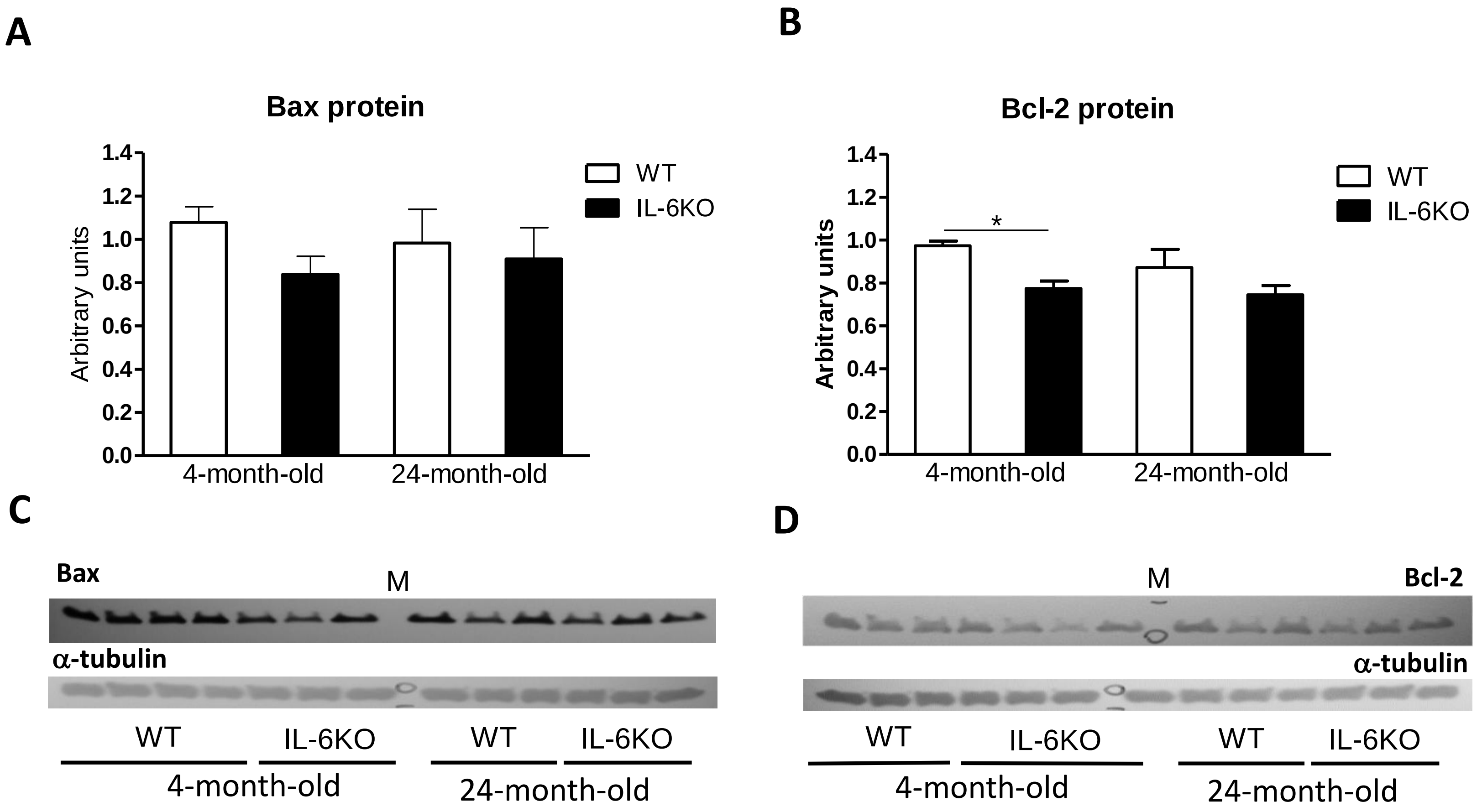

Fig. S2 Pro-apoptotic Bax protein (**A**) and anti-apoptotic Bcl-2 protein (**B**) in hippocampus of 4- and 24-month-old IL-6-deficient (IL-6KO) and wild type control (WT) mice. Bars represent mean ± SEM obtained from 6 animals in each group. There were no significant differences in the amount of Bax in tested groups of mice. Analysis of Bcl-2 protein expression revealed its lower levels in IL-6KO mice, which was statistically significant in young adult group ($p < 0.05$, ANOVA and Bonferroni post-hoc). Aging did not affect expression of Bcl-2 protein. (**C, D**) Representative immunoblots, for Bax and Bcl-2 proteins, respectively, are shown together with α-tubulin as a loading control. M - molecular weight marker
